# Clinical and microbiological evaluation of temocillin for bloodstream infections with Enterobacterales: a Belgian single-centre retrospective study

**DOI:** 10.1093/jacamr/dlac086

**Published:** 2022-08-23

**Authors:** Julie Oosterbos, Maaike Schalkwijk, Steven Thiessen, Els Oris, Guy Coppens, Katrien Lagrou, Deborah Steensels

**Affiliations:** Ziekenhuis Oost-Limburg hospital, Genk, Belgium; Ziekenhuis Oost-Limburg hospital, Genk, Belgium; Ziekenhuis Oost-Limburg hospital, Genk, Belgium; Ziekenhuis Oost-Limburg hospital, Genk, Belgium; Ziekenhuis Oost-Limburg hospital, Genk, Belgium; Katholieke Universiteit Leuven, Leuven, Belgium; Ziekenhuis Oost-Limburg hospital, Genk, Belgium; Université Libre de Bruxelles, Brussels, Belgium

## Abstract

**Background:**

Expanding the use of temocillin could be an important weapon in the fight against antimicrobial resistance. However, EUCAST defined clinical breakpoints for a limited number of species and only for urinary tract infections (UTI), including urosepsis but excluding severe sepsis and septic shock. Moreover, a dosage of 2 g q8h is advised in most cases.

**Objectives:**

Evaluation of temocillin use for the treatment of bacteraemia, correlating clinical and microbiological outcomes with infection site, infection severity, temocillin dosage, Enterobacterales species and MIC.

**Patients and methods:**

All adult patients with blood cultures positive for temocillin-susceptible Enterobacterales and treated with temocillin for ≥72 h from June 2018 until June 2021 were considered for inclusion. The primary outcome was clinical success, defined as resolution of infection signs, no relapse of the same infection and no antibiotic switch due to insufficient clinical improvement. The secondary outcome was microbiological success.

**Results:**

In total, 182 episodes were included [140 UTI versus 42 non-UTI, 171 *Escherichia coli, Klebsiella* species (except *Klebsiella aerogenes*) and *Proteus mirabilis* (EKPs) versus 11 non-EKPs]. Clinical and microbiological failure were low (8% and 3%, respectively). No difference in outcome was observed for dosages of 2 g q12h versus 2 g q8h, either for EKP versus non-EKP isolates or MIC values ≤8 versus 16 mg/L. Considering only bacteraemia episodes of UTI origin, using the 16 mg/L breakpoint, there was no difference in success rate between regimens of 2 g q12h and 2 g q8h.

**Conclusions:**

Temocillin 2 g q12h can be successfully used for the treatment of systemic UTI. Prospective studies are needed to assess outcomes and evaluate non-inferiority compared with other broad-spectrum antibiotics in non-UTI infections, including bacteraemia.

## Introduction

While broad-spectrum antibiotics play an invaluable role in the treatment of bacterial infections, they have a high propensity for bacterial resistance selection and a detrimental effect on the host microbiome. With the rising threat of MDR microorganisms, giving preference to the use of narrow-spectrum alternatives is desirable.^1,2^

Temocillin (BRL 17421), the 6-α-methoxy derivative of ticarcillin, is a narrow-spectrum IV β-lactam antibiotic with activity against many Gram-negative bacteria. It has no activity for Gram-positive bacteria, anaerobes or *Pseudomonas aeruginosa*.^3^ Thanks to the methoxy group, temocillin is stable against hydrolysis of many ESBL–producing Enterobacterales.^4^

In Belgium, temocillin is indicated for the treatment of the following infections in adults and children, where susceptible Gram-negative bacilli are highly suspected or confirmed: complicated urinary tract infections (UTI) including acute pyelonephritis; lower respiratory tract infections (including nosocomial pneumonia); acute infections of the skin and soft tissues; and bacteraemias associated or suspected to be related to any of these infections.^5^ Currently, temocillin is only licensed in Belgium, France, the UK, Germany, Luxembourg and Iran.^6,7^

In 1985, Fuchs *et al.*^8^ published the first interpretive criteria for temocillin susceptibility testing (Table 1). Until recently, only BSAC and the Comité de l’antibiogramme de la Société Française de Microbiologie (CA-SFM) published breakpoints for temocillin.^9,10^ Since April 2020, EUCAST has published off-scale clinical breakpoints, valid for a limited number of species and only for UTI, including urosepsis but excluding severe sepsis and septic shock.^11–13^ All strains are categorized as ‘susceptible, increased exposure (I)’ for an MIC value up to 16 mg/L, meaning a dose of 2 g q8h is required. EUCAST however states the standard dose of 2 g q12h has to be used for uncomplicated UTI only. EUCAST defines uncomplicated UTI as acute, sporadic or recurrent lower UTI (uncomplicated cystitis) in patients with no known relevant anatomical or functional abnormalities within the urinary tract or comorbidities.^12^

**Table 1.** Overview of MIC value and disc diffusion breakpoints for temocillin

Breakpoint	Fuchs *et al.* (1985)		BSAC (2013)		CA-SFM (2019)		EUCAST (2022)	
	S	R	S	R	S	R	S	R
MIC value (mg/L)	≤16	≥32	Urinary: ≤32	Urinary: >32	≤8	>8	≤0.001	>16
			Systemic: ≤8	Systemic: >8				
Disc diffusion (mm), 30 μg	≥19	≤15	Urinary: ≥12	Systemic: ≤11	≥20	<20	≥50	<17
			Systemic: ≥20	Systemic: ≤19				

S, susceptible; R, resistant.

EUCAST also states that temocillin ‘is used principally to treat more serious infections caused by Enterobacterales producing extended-spectrum and AmpC type β-lactamases.’ This is not in line with Belgian clinical practice, where temocillin has been used for several decades and where it is recommended by the Belgian Society of Infectious disease and Clinical Microbiology (BVIKM) as a first-line treatment of several urinary tract infections.^14^ In our hospital, Ziekenhuis Oost-Limburg (ZOL), temocillin has been used for more than 20 years. It is prescribed empirically in UTI and urosepsis (including severe sepsis and septic shock), but also in serious (early-onset) hospital-acquired pneumonias (in combination with flucloxacillin) and is used as directed therapy for a broad range of indications (including bacteraemia) to spare broad-spectrum antibiotics. In this retrospective study, we aimed to evaluate temocillin use for the treatment of bacteraemia with Enterobacterales, correlating clinical and microbiological outcomes to the infection site, severity of the bacteraemia, temocillin dosage, Enterobacterales species and MIC value.

## Patients and methods

### Patient population

A single-centre retrospective observational analysis was performed in ZOL, a tertiary care centre in Belgium. During a 3 year period (June 2018 until June 2021), all adult patients having positive blood cultures for temocillin susceptible Enterobacterales and treated with temocillin for at least 72 h were included.

### Temocillin dosage

In ZOL, for patients with a normal renal function, a dosage of 2 g q12h is recommended for uncomplicated UTI (definition EUCAST) and a dosage of 2 g q8h for all other indications.^12^ Up until December 2019 however, even for complicated UTI (definition EUCAST) with or without bacteraemia, the lower dosage of 2 g q12h was prescribed.^12^ Dosages are adjusted to renal function according to the local hospital guidelines (Table 2), in turn based on the IGGI guidelines from the BVIKM,^14^ and adjusted to the EUCAST guidelines for standard dosage and high dosage.^11,12^ Patients receiving dosages of (or equivalent to after correction for renal function) 2 g q12h and 2 g q8h were included. Patients who received equivalent dosages less than 2 g q12h or greater than 2 g q8h were excluded.

**Table 2.** Temocillin dosage according to renal function

Indication	First dose/loading dose	eGFR (mL/min/1.73 m^2^)					IHD	CVVH
		>90	60–89	30–59	15–29	<15		
Urinary tract infection and not septic^16^	2 g	2 g q12h	2 g q12h	2 g q12h	2 g q24h	1 g q24h	1 g q24h	2 g q12h
							Supplemental dose *after* dialysis: 2 g	
Septic and/or non-urinary tract infection^16^	2 g	2 g q8h	2 g q8h	2 g q12h	2 g q24h	1 g q24h	1 g q24h	2 g q12h
							Supplemental dose *after* dialysis: 2 g	

IHD, intermittent haemodialysis; CVVH, continuous venovenous haemofiltration.

### Susceptibility testing

Temocillin susceptibility was determined by both disc diffusion (Liofilchem, Italy) and Etests (Liofilchem). Breakpoints described by Fuchs *et al.*^8,15^ were applied for susceptibility reporting. Disc diameters were read by the automatic system SIRscan 2000®, with phenotypic detection of ESBL and AmpC resistance profiles. When an ESBL was detected, a confirmation test was performed using the double-disc synergy test.

### Clinical and microbiological outcome parameters

Demographic and clinical parameters, such as underlying diseases; infection site; severity of bacteraemia (septic shock versus non-septic shock);^16^ C-reactive protein (CRP); temperature; antibiotic treatment; observed side effects (if described); and ICU admission (between 7 days prior to 7 days after initiation of temocillin treatment) were collected from the electronic patient records.

The primary outcome was clinical success, defined as resolution of infection signs, no relapse of the same infection within hospitalization and no switch of antibiotics due to insufficient clinical improvement. Clinical failure was defined by switch to other (mostly broad-spectrum) antibiotics due to insufficient clinical improvement in combination with persistence of infection signs or relapse of the same infection. Increased or pending CRP (if assessable) and persistent fever (if assessable) were taken into account likewise. When clinical failure was rated, additional information about source control, foreign material, immune status, comorbidities and additional infections was collected. Cases where antibiotics were switched due to an additional, superposing infection for which temocillin was not suitable were excluded. Cases where empirical therapy (with activity for the isolated microorganism) was given more than 72 h and switches to oral antibiotics after less than 72 h were also excluded. Finally, the transition to a palliative setting, independent of the bacteraemia, was also an exclusion criterion.

The secondary outcome was microbiological success, defined as the absence of a breakthrough bacteraemia (defined as the presence of positive blood cultures at least 48 h after the previous positive blood cultures under adequately dosed therapy) and no relapse of the same bacteraemia within 14 days.^17,18^ According to the local hospital guidelines, repeated blood cultures were taken when symptoms persisted and when clinical failure was suspected. In cases with microbiological failure, additional information about source control, foreign material, immune status, comorbidities and additional infections was collected.

Cases with (possible) clinical and/or microbiological failure were evaluated by a second specialist (infectiologist) to ensure unequivocal categorization. Besides these two endpoints, all-cause mortality within 28 days (in-hospital mortality), adverse effects and *Clostridioides difficile* infections (defined as laboratory proven toxin-producing species) were evaluated. Finally, the outcome parameters were correlated to patients’ demographics and clinical features, severity of the bacteraemia (septic shock versus non-septic shock), type of Enterobacterales species, MIC values, temocillin dosages and infectious sites.

### Statistical evaluation

Statistical analyses were performed with JMP PRO version 16.0 (SAS Institute Inc., Cary, NC, USA). For continuous data, parametric comparisons between groups were made with Student’s *t*-test for normally distributed data. For categorical data, Pearson’s χ^2^ test was used. A (two-sided) *P* value of 0.05 or lower was considered statistically significant.

### Study approval

The study was approved by the Medical Ethics Committee of ZOL (internal reference number CTU2020111).

## Results

**Figure 1.**
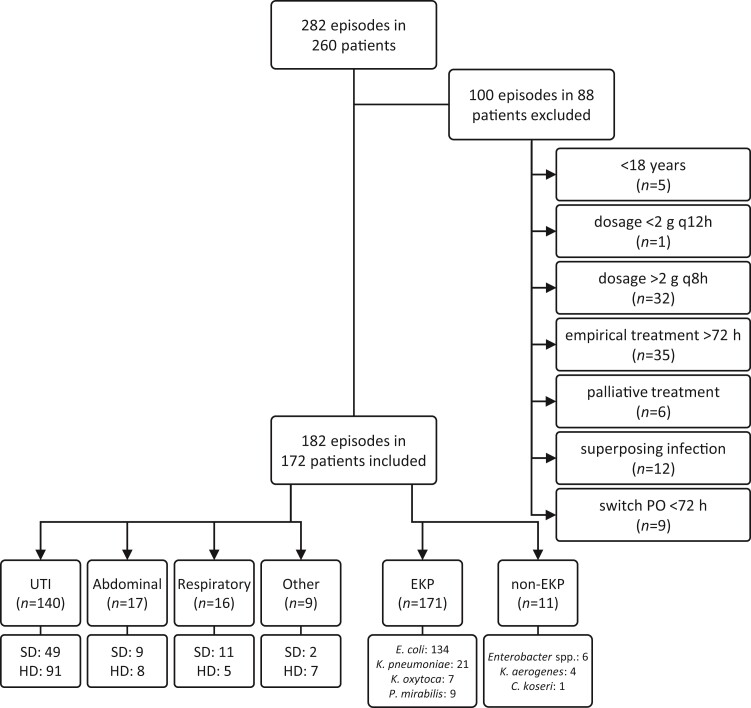
Overview of study population. SD, standard dosage, equivalent to 2 g q12h; HD, high dosage, equivalent to 2 g q8h.

In total, 282 episodes of bacteraemia with Enterobacterales were evaluated, of which 182 were included. The main reasons for exclusion were the reception of other empirical antibiotics for more than 72 h (*n* = 35) or higher equivalent dosages than defined (*n* = 32) (Figure 1). Men and women were approximately equally represented, with a mean age of 74 years (Table 3). The infectious sites of the bacteraemia were dominantly UTI (*n* = 140), followed by intra-abdominal infections (*n* = 17) and respiratory infections (*n* = 16). In 66% of the episodes, temocillin was started empirically. In other episodes, empirical therapy was switched to temocillin based on susceptibility testing. *Escherichia coli, Klebsiella* species (except *Klebsiella aerogenes*) and *Proteus mirabilis* (EKP) isolates were clearly in the majority (*n* = 171), with a preponderance of *E. coli* isolates (*n* = 134) (Figure 1). In total, 32 ESBLs were identified and four AmpC producers. Overall, 39% of the patients received a dose equivalent to 2 g q12h and 61% a dose equivalent to 2 g q8h (Table 3). Temocillin was generally administered via intermittent infusion, with the exception of patients in the ICU who received temocillin via continuous infusion.

**Table 3.** Patient demographics and clinical characteristics according to clinical outcome and microbiological outcome

Characteristics	All (*n* = 182)	Clinical success (*n* = 167, 92%)	Clinical failure (*n* = 15, 8%)	*P*	Microbiological success (*n* = 177, 97%)	Microbiological failure (*n* = 5, 3%)	*P*
Patients							
Age (years), mean ± SD	74 ± 12	74 ± 12	70 ± 12	0.21	74 ± 12	67 ± 11	0.20
Sex, *n* (%)				0.22			0.15
Male	94 (52)	84 (89)	10 (11)		93 (99)	1 (1)	
Female	88 (48)	83 (94)	5 (6)		84 (95)	4 (5)	
Charlson Comorbidity Index, mean ± SD	5.25 ± 2.61	5.17 ± 2.53	6.07 ± 3.35	0.33	5.23 ± 2.60	5.80 ± 3.27	0.72
Diabetes mellitus, *n* (%)				0.73			0.31
Yes	30 (16)	28 (93)	2 (7)		30 (100)	0 (0)	
No	152 (84)	139 (91)	13 (9)		147 (97)	5 (3)	
Chronic kidney disease, *n* (%)				0.37			0.89
Yes	41 (23)	39 (95)	2 (5)		40 (98)	1 (2)	
No	141 (77)	128 (91)	13 (9)		137 (97)	4 (3)	
Immunosuppression, *n* (%)				**0.01**			0.15
Yes	30 (16)	24 (80)	6 (20)		28 (93)	2 (7)	
No	152 (84)	143 (94)	9 (6)		149 (98)	3 (2)	
Intensive care, *n* (%)				** < 0.01**			** < 0.01**
Yes	24 (13)	16 (67)	8 (33)		21 (88)	3 (12)	
No	158 (87)	151 (96)	7 (4)		156 (99)	2 (1)	
All-cause mortality, *n* (%)	12 (7)	5 (42)	7 (58)	**<0**.**01**	10 (83)	2 (17)	**<0**.**01**
Infection							
Severity, *n* (%)				**<0.01**			**0**.**01**
No septic shock	166 (91)	157 (95)	9 (5)		163 (98)	3 (2)	
Septic shock	16 (9)	10 (63)	6 (37)		14 (88)	2 (12)	
Onset, *n* (%)				**0.03**			**0.05**
Community acquired	139 (76)	131 (94)	8 (6)		137 (99)	2 (1)	
Hospital acquired	43 (24)	36 (84)	7 (16)		40 (93)	3 (7)	
Infectious site, *n* (%)				**<0.01**			**<0**.**01**
UTI	140 (77)	137 (98)	3 (2)		140 (0)	0 (0)	
Non-UTI	42 (23)	30 (71)	12 (29)		37 (88)	5 (12)	
Microbiological characteristics							
Enterobacterales species, *n* (%)				0.92			0.18
EKP	171 (94)	157 (92)	14 (8)		167 (98)	4 (2)	
Non-EKP	11 (6)	10 (91)	1 (9)		10 (91)	1 (9)	
MIC value (mg/L), *n* (%)				0.42			0.38
1 to ≤8	158 (87)	146 (92)	12 (8)		153 (97)	5 (3)	
16	24 (13)	21 (88)	3 (12)		24 (100)	0 (0)	
Resistance profile, *n* (%)				**0.04**			**0**.**02**
ESBL- or AmpC-producing	36 (20)	30 (83)	6 (17)		33 (92)	3 (8)	
No ESBL or AmpC detected	146 (80)	137 (94)	9 (6)		144 (99)	2 (1)	
Temocillin regimen							
Duration (days), median (IQR)	6 (4–9)	6 (4–9)	10 (7–11)	**0**.**05**	6 (4–9)	10 (10–11)	**0**.**01**
Dosage				0.64			0.96
Equivalence 2 g q12h	71 (39)	66 (93)	5 (7)		69 (97)	2 (3)	
Equivalence 2 g q8h	111 (61)	101 (91)	10 (9)		108 (97)	3 (3)	
Prior (empirical) antibiotic therapy with activity, *n* (%)				0.94			0.76
Yes	47 (26)	43 (91)	4 (9)		46 (98)	1 (2)	
No	135 (74)	124 (92)	11 (8)		131 (97)	4 (3)	
Concomitant antibiotic therapy with activity, *n* (%)				**0.05**			**0**.**04**
Aminoglycosides	80 (44)	77 (96)	3 (4)		80 (100)	0 (0)	
No aminoglycosides	101 (56)	89 (88)	12 (12)		96 (95)	5 (5)	

*P* values ≤0.05 are indicated in bold.

### Clinical and microbiological outcome

Overall, clinical and microbiological success was observed in 92% and 97% of episodes, respectively. Table 3 shows the collected variables for the included bacteraemia episodes altogether as well as split by clinical and microbiological success and failure groups. Age, sex, Charlson Comorbidity Index, diabetes mellitus and chronic kidney disease were not different between success and failure groups. However, the presence of underlying immune suppression was higher in the clinical failure group (*P* = 0.01) (Table 3).

The use of (mostly β-lactam) antibiotics prior to temocillin therapy (≤72 h) was comparable for success and failure groups. More clinical and microbiological success was seen in the group with concomitant therapy with aminoglycosides (respectively *P* = 0.05 and *P* = 0.04). Duration of temocillin therapy was longer in the group with microbiological failure (*P* = 0.01) and clinical failure (*P* = 0.05). Interestingly, there was no difference in clinical and microbiological success rate between temocillin dosage regimens of 2 g q12h and 2 g q8h (Table 3, 93% versus 91% for clinical success and 97% versus 97% for microbiological success, respectively). When considering only bacteraemia episodes with a UTI origin, there was also no difference in success rate between temocillin dosage regimens of 2 g q12h versus 2 g q8h (*P* = 0.20). Since temocillin dosing overlaps between the 2 g q12h and 2 g q8h regimen in case of impaired renal function, the effect of dosing regimen on outcome was analysed for the subgroup of patients with an eGFR of >60  mL/min (49%). For this subgroup, there was even a trend towards a higher success rate for the 2 g q12h regimen (*P* = 0.77 for microbiological outcome, *P* = 0.46 for clinical outcome). In addition, there was no difference in clinical and microbiological outcome in episodes caused by strains with an MIC value ≤8 mg/L versus those with an MIC of 16 mg/L. Important to note, there was no difference in dosing regimens between these groups (*P* = 0.29).

For patients who required intensive care, the clinical and microbiological failure rates were higher (*P* < 0.01). Consistently, presence of septic shock was significantly higher in patients with clinical and microbiological failure (*P* < 0.01 and *P* = 0.01, respectively) (Table 3).

In the group of episodes with a non-UTI focus, clinical and microbiological failure rates were also higher (*P* < 0.01). However, ICU admission, immunosuppression and septic shock were significantly higher in this non-UTI group (*P* < 0.01). The association of hospital-acquired infections with more clinical and microbiological failure compared with community-acquired infections (Table 3) was also biased by a difference in ICU admission and septic shock between these groups (*P* < 0.01).

The type of Enterobacterales species was not significantly different between success and failure groups (Table 3). The presence of an ESBL or AmpC resistance mechanism was higher in the clinical and microbiological failure groups (*P* = 0.04 and *P* = 0.02, respectively). However, the presence of ESBL or AmpC resistance was associated with a higher incidence of an immunosuppressed state (*P* = 0.04).

As expected, all-cause mortality was higher in clinical and microbiological failure groups (Table 3).

### Clinical failure group

In total, 15 bacteraemia episodes under temocillin therapy (8%) (in 14 patients) showed an unfavourable clinical outcome (Table S1 for detailed description, available as Supplementary data at *JAC-AMR* Online). Four cases were clearly attributable to the lack of source control. Two cases were attributed to underlying comorbidities of the patient. The other nine bacteraemia episodes (in eight patients) with clinical failure could possibly be due to failure of temocillin treatment. The focus of the bacteraemia was respiratory in five patients, intra-abdominal in one patient and urinary in two patients. All eight patients had other contributing factors for clinical failure, and four patients were critically ill needing intensive care. Nevertheless, in five cases, a switch after 5 to 13 days of temocillin to meropenem or ciprofloxacin led to clinical improvement.

### Microbiological failure group

In total, five bacteraemia episodes under temocillin therapy (3%) showed microbiological failure, all within the group with clinical failure. All were non-UTI bacteraemias. Three cases were defined as breakthrough bacteraemias, two cases as the same episode of bacteraemia within 2 weeks. An increase of MIC value towards resistance was seen in four out of five cases. Four isolates belonged to the EKP group, one to the non-EKP group. Two strains were positive for ESBL and one for AmpC resistance.

### All-cause mortality

Of all included patients, 11/172 (6%) died within 28 days of their positive blood cultures. Six of them were categorized in the clinical failure group, of which two had microbiological failure. All patients had non-UTI bacteraemias with Enterobacterales EKP group. One episode was caused by an ESBL-producing strain. Three patients were admitted to the ICU, of which two had septic shock. Underlying comorbidities that contributed to their death were present in each case.

### Adverse events

No adverse events for temocillin treatment were recorded.

### C. difficile infections

Only two patients (1.2%) were suffering from *C. difficile*-associated diarrhoea. One patient received another β-lactam antibiotic prior to temocillin therapy. The other patient tested positive at Day 5 of the temocillin therapy. However, at the time of admission, symptoms of diarrhoea were already present and no tests were performed. In both cases, the infection could not be directly attributed to treatment with temocillin.

## Discussion

First of all, clinical and microbiological failure rates in patients treated with temocillin for bacteraemias with temocillin- susceptible Enterobacterales were low (8% and 3%, respectively). Failure could not be attributed to an insufficient treatment duration.

The effectiveness of temocillin treatment for UTI with or without bacteraemia is well established.^19–21^ Our results confirm a high clinical success rate (98%) for UTI with bacteraemia. Importantly, similar outcome data were found between the 2 g q12h temocillin group and the 2 g q8h group. However, when following EUCAST guidelines, a dosage of 2 g q12h should only be used for the treatment of uncomplicated UTI, excluding all systemic infections.^22^ They state the standard dosage of 2 g q12h provides insufficient drug exposure to cover all infecting strains, even when these are without acquired resistance mechanisms. EUCAST mentions the standard dose will only provide sufficient drug exposure in 70% to 80% of patients, even for systemic infections originating in the urinary tract. They aim for a coverage of 95% to 99% of patients.^22^ Since temocillin is an expensive drug, the difference in cost per treatment day is significant between 2 g q12h and 2 g q8h.^23^ Also, prescribing 2 g q8h might limit outpatient therapy.

Several studies evaluated the use of temocillin for the treatment of non-UTI, however proof of non-inferiority compared with other antibiotics remains scarce in this group.^21,24–27^ Prospective randomized control trials are needed, including the different dosing regimens for temocillin. Currently, a few clinical trials are registered comparing temocillin with a carbapenem for treatment of ESBL and/or AmpC infections [for example the TEMO-CARB (NCT03543436), TEMO-BLSE (NCT04671290) and TEMO-ESBL (NCT02681263) clinical trials, which are in either recruitment or completion phases].^28^ In our study, non-UTI focus was associated with more clinical and microbiological failure. However, patients with a non-UTI origin were significantly more immunosuppressed and were more severely ill as compared with patients suffering from a UTI origin. Therefore, the increased failure in this group might be secondary to the condition of the patient instead of the failure of the antibiotic treatment with temocillin.

As to be expected, our study results showed more clinical and microbiological failure and a higher mortality in patients with ICU admission and septic shock. Critically ill patients with Gram-negative bacteraemia are predisposed to have worse outcomes, regardless of the antibiotic treatment used. Therapy efficacy can potentially be reduced due to pharmacokinetic and pharmacodynamic alterations. This might be overcome by the administration of 6 g temocillin via continuous infusion, as described by Laterre *et al.*^12,26^ Nonetheless, EUCAST guidelines do not support the use of temocillin for treatment of infections with severe sepsis and septic shock. Prospective studies comparing temocillin with other antibiotics for these indications are needed.

EUCAST does not provide breakpoints for non-EKP isolates because of an insufficient number of acceptable distributions to determine epidemiological cut-off values (ECOFFs). In addition, it is known that acquired temocillin resistance could be a problem for *Enterobacter* species and *Serratia marcescens*. This was already described by Fuchs *et al.* in 1984 and is mentioned in the leaflet of Negaban®.^5,15^ However, we did not find a significant difference in outcomes between episodes with EKP and non-EKP isolates. These findings are in line with the study results of Balakrishnan *et al.*^19^ and Alexandre *et al.*^20^ More large-scale clinical studies are needed to substantiate our findings and to determine whether a clinical breakpoint can be set for non-EKP isolates.

*In vitro* activity studies have set an MIC_90_ of 16 mg/L for Enterobacterales, except for *Enterobacter* species and *S. marcescens* where higher MIC_90_ values were observed.^15,28,29^ Based on pharmacokinetic studies using Monte Carlo simulations performed on ICU patients, a breakpoint of 8 mg/L was proposed for the standard dosage of 2 g q12h and a breakpoint of 16 mg/L for the high dosage of 2 g q8h.^26,30^ When comparing the outcomes of episodes caused by strains with an MIC value ≤8 mg/L versus those with an MIC of 16 mg/L, we did not observe a significant difference. Since there was no difference in dosing regimens between the groups with MIC values ≤8 mg/L and 16 mg/L, our results indicate that the breakpoint of 16 mg/L proposed by Fuchs *et al.*^8^ might safely be used for both dosing regimens, at least in non-critically ill patients and UTI episodes.

Finally, temocillin was very well tolerated by all included patients. No side effects were registered, neither was there a causative link with subsequent *C. difficile* infections. These findings are in line with other literature. In addition, temocillin has no effect on colonization resistance.^27,31–33^

Limitations of this study include the retrospective nature and the lack of comparison with another antibiotic drug. It would be interesting to match the outcome results to those of patients who received piperacillin/tazobactam, a carbapenem or another broad-spectrum antibiotic for the same indication. Also, it was not possible to assess for presence of sepsis or severe sepsis retrospectively, so the severity of infection could only be differentiated as presence or absence of septic shock. In addition, temocillin was commonly switched to oral antibiotics after 3 or 4 days of therapy. Due to the retrospective nature of this study, the timing of switch was not standardized. In addition, patients were often discharged before completion of oral therapy, so we were not always able to assess outcomes until the end of therapy. Nevertheless, the study design does allow for evaluation of temocillin efficacy during the acute phase of bacteraemia. Of note, the local dosing guidelines were not always correctly followed by the prescribers, resulting in the majority of abdominal and respiratory infections being treated with the lower dose.

In conclusion, this observational retrospective study adds to the knowledge on temocillin efficacy for the treatment of bacteraemia and in particular on the comparison of different dosing regimens. Low clinical and microbiological failure rates were found for the treatment of bacteraemias with temocillin-susceptible Enterobacterales while using the Fuchs *et al.*^8^ breakpoint of 16 mg/L. Clinical and microbiological failure was mostly attributable to the patient’s degree of illness, underlying comorbidities or source control. No difference in outcome was observed for dosages of 2 g q12h versus 2 g q8h, either for EKP versus non-EKP isolates or MIC values ≤8 mg/L versus 16 mg/L. For treatment of systemic UTI, using the standard dosage of temocillin seems sufficient. Since temocillin is an important weapon in the fight against antimicrobial resistance, research on temocillin efficacy for as many indications as possible is important.

## Supplementary Material

dlac086_Supplementary_Data

## Supplementary data

Table S1 is available as Supplementary data at *JAC-AMR* Online.
