# Supplementary material for: Clinical and microbiological evaluation of temocillin for bloodstream infections with Enterobacterales: a Belgian single-centre retrospective study
Source: JAC Antimicrob Resist. 2022 Aug 23;4(4):dlac086. doi: 10.1093/jacamr/dlac086 (PMC9397121; doi:10.1093/jacamr/dlac086)
Supplement: dlac086_Supplementary_Data [file dlac086_supplementary_data.docx]

**Supplementary data**

**Table S1**. Detailed description of bacteraemia cases with clinical failure

| **Patient** | **Site of infection** | **Dosage** | **Onset** | **ICU** | **Septic shock** | **Immune suppression** | **Presumed reason of failure** | **Detail** |
| --- | --- | --- | --- | --- | --- | --- | --- | --- |
| 1 | Respiratory | Equiv.  2g q12h | HA | Yes | Yes | No | Failure under temocillin | Recurrent respiratory infections with bacteraemia and haematogenic spread (thrombophlebitis) after intracranial haemorrhage. |
| 2 | Abdominal | Equiv.  2g q8h | CA | No | No | Yes | Failure under temocillin | Abdominal infection with breakthrough bacteraemia in a patient with metastatic pancreatic cancer. Slight clinical improvement after switch to meropenem, after which transferred to the palliative care unit. |
| 3 | Abdominal | Equiv.  2g q8h | CA | Yes | Yes | No | Source control | Septic shock post bile leakage and small bowel ischemia secondary to abdominal compartment syndrome where no surgical intervention was possible. |
| 4 | Respiratory | Equiv.  2g q12h | HA | Yes | Yes | Yes | Comorbidities patient | Patient on ECMO (extracorporeal membrane oxygenation) with complicated COVID-19 infection. Multi organic failure after weaning with evolution to cardiac arrest. |
| 5 | Urinary | Equiv.  2g q8h | CA | Yes | No | No | Failure under temocillin | Urinary infection with bacteraemia in patient with Bricker bladder and renal cysts. Persistent fever and CRP under temocillin treatment, significant clinical improvement when switched to ciprofloxacin. |
| 6 | Respiratory | Equiv.  2g q8h | HA | Yes | Yes | No | Failure under temocillin | COVID-19 pneumonia with breakthrough bacteraemia. Clinical improvement after switch to ciprofloxacin. |
| 7 | Respiratory | Equiv.  2g q8h | HA | Yes | Yes | No | Comorbidities patient | Post CABG respiratory infection with bacteraemia. Sudden deterioration with multi organ failure and shock. |
| 8 | Respiratory | Equiv.  2g q12h | HA | Yes | Yes | No | Failure under temocillin | Ventilator-associated pneumonia (VAP) with bacteraemia. Clinical improvement after switch to ciprofloxacin. |
| 9 | Respiratory | Equiv.  2g q8h | HA | No | No | No | Failure under temocillin | Respiratory infection with breakthrough bacteraemia after craniocerebral trauma. Clinical improvement after switch to meropenem and catheter change. |
| 10 | Abdominal | Equiv.  2g q12h | CA | Yes | No | Yes | Source control | Complicated abdominal infection with breakthrough bacteraemias caused by insufficient source control. Development of small bowel obstruction post-ERCP (endoscopic retrograde cholangiopancreatography) with no clinical improvement after switch to different broad-spectrum antibiotics. |
| 11 | Abdominal | Equiv.  2g q12h | HA | No | No | Yes | Source control | Complicated abdominal infection with multiple abscesses and fistulas where no chirurgical intervention was possible. |
| 12 | Urinary | Equiv.  2g q8h | CA | No | No | No | Source control | Urinary bacteraemia with insufficient clinical improvement caused by not changing the urinary catheter. |
| 13 | Respiratory | Equiv.  2g q8h | CA | No | No | Yes | Failure under temocillin | Respiratory infection in patient with an extensive pulmonary medical history. No improvement was seen under temocillin therapy. Eventually, patient was transferred to the palliative care unit. |
| 14 | Urinary | Equiv.  2g q8h | CA | No | No | No | Failure under temocillin | Patient with neobladder and suprapubic probe. Multifocal pyelonephritis with gradual clinical improvement after switch to meropenem. |
